# An Analysis of Peer-Reviewed Scores and Impact Factors with Different Citation Time Windows: A Case Study of 28 Ophthalmologic Journals

**DOI:** 10.1371/journal.pone.0135583

**Published:** 2015-08-21

**Authors:** Xue-Li Liu, Shuang-Shuang Gai, Shi-Le Zhang, Pu Wang

**Affiliations:** 1 Periodicals Publishing House, Xinxiang Medical University, Xinxiang, Henan Province, China; 2 Henan Research Center for Science Journals, Xinxiang Medical University, Xinxiang, Henan Province, China; 3 Xiamen University Tan Kan Kee College Library, Zhangzhou, Fujian Province, China; Mälardalen University, SWEDEN

## Abstract

**Background:**

An important attribute of the traditional impact factor was the controversial 2-year citation window. So far, several scholars have proposed using different citation time windows for evaluating journals. However, there is no confirmation whether a longer citation time window would be better. How did the journal evaluation effects of 3IF, 4IF, and 6IF comparing with 2IF and 5IF? In order to understand these questions, we made a comparative study of impact factors with different citation time windows with the peer-reviewed scores of ophthalmologic journals indexed by Science Citation Index Expanded (SCIE) database.

**Methods:**

The peer-reviewed scores of 28 ophthalmologic journals were obtained through a self-designed survey questionnaire. Impact factors with different citation time windows (including 2IF, 3IF, 4IF, 5IF, and 6IF) of 28 ophthalmologic journals were computed and compared in accordance with each impact factor’s definition and formula, using the citation analysis function of the Web of Science (WoS) database. An analysis of the correlation between impact factors with different citation time windows and peer-reviewed scores was carried out.

**Results:**

Although impact factor values with different citation time windows were different, there was a high level of correlation between them when it came to evaluating journals. In the current study, for ophthalmologic journals’ impact factors with different time windows in 2013, 3IF and 4IF seemed the ideal ranges for comparison, when assessed in relation to peer-reviewed scores. In addition, the 3-year and 4-year windows were quite consistent with the cited peak age of documents published by ophthalmologic journals.

**Research Limitations:**

Our study is based on ophthalmology journals and we only analyze the impact factors with different citation time window in 2013, so it has yet to be ascertained whether other disciplines (especially those with a later cited peak) or other years would follow the same or similar patterns.

**Originality/ Value:**

We designed the survey questionnaire ourselves, specifically to assess the real influence of journals. We used peer-reviewed scores to judge the journal evaluation effect of impact factors with different citation time windows. The main purpose of this study was to help researchers better understand the role of impact factors with different citation time windows in journal evaluation.

## Introduction

Thomson Reuters announced the launch of an enhanced edition of Journal Citation Reports (JCR) on January 22, 2009 [1–2]. This enhanced JCR updated the 2007 edition (JCR-2007) released in July 2008 in part by adding three important bibliometric indicators: the Eigenfactor Score (EFS), the Article Influence Score (AIS) and the 5-year Impact Factor (5IF). 5IF was an important supplement to the traditional 2-year Impact Factor (2IF), the only distinction between them being the different citation time windows or timeframes used to calculate the impact factor (the former using a 5-year citation time window and the latter a 2-year citation time window) [3–4].

Some scholars [5] objected to the use of a 2-year citation time window for calculating impact factors as early as 1997, arguing that while a 2-year citation time window was suitable for some subjects, it was not suitable for others. Most scholars believed that the citation time used for calculating 2IF was too short and lacked statistical rationality [4, 6, 7, 8]. The 5IF emerged from just such a background. The 5IF has attracted widespread concern from scholars around the world [9–15] ever since it became a JCR journal evaluation indicator. However, would a longer citation time window be better? How did the journal evaluation effects of 3IF, 4IF, and 6IF comparing with 2IF and 5IF? In order to understand these questions, we made a comparative study of impact factors with different citation time windows, using ophthalmologic journals indexed by SCIE as our case studies.

## Literature Review

### Journal impact factor

It has been over fifty years since the concept of impact factor was first proposed by Garfield, a famous American bibliometric expert, in 1955 [16–18]. In 1963, Garfield and Sher proposed using the concept of impact factor to re-evaluate the influence of journals [19]; Garfield officially established a precise concept and method of computing impact factor in 1972 [20]. Impact factor became a real bibliometric indicator for scientific journal evaluation in 1975, when the JCR database was built. Now confirmed as an important journal evaluation tool, the concept of impact factor has attracted widespread interest and been widely applied [21–24]. However, its increasing popularity application as a tool for journal evaluation has revealed many major defects of the impact factor concept. Its main flaws have been identified as follows:

1. The two-year citation window used to calculate impact factor is too short to evaluate the real impact of publications especially those with a later cited peak as occur in many social sciences in which maturation time of citations is slower [25–26].
2. “Journal impact factor,” as defined by the JCR database, does not exclude self-citation; this oversight has enabled many journals to manipulate their impact factor levels by improving self-citations.
3. Although the numerator in the impact factor calculation was the total number of citations in a statistical year for all documents published within the previous two years, the denominator only included articles and reviews published during the previous two years.
4. The method of computing impact factor did not accord with statistical principles. Citations of papers did not fit into normal distribution. As all were aware, when data does not fit normal distribution patterns, the mean is not a good parameter to describe the distribution of citations—despite this, journal impact factor was the average number of citations in a statistical year for papers published within the previous two years.
5. The impact factor couldn’t be used to evaluate journals across disciplines, which greatly limited its value.

As the use of impact factor in journal evaluation rapidly increased, debates about its various shortcomings became more and more fierce. Although the debate about the validity of impact factor in journal evaluation has never been fully resolved, the current study focuses mainly on the problem of the citation time window in assessing impact factor.

### Citation time window of impact factor

An important attribute of the traditional impact factor was the controversial 2-year citation window developed by Martyn and Gilchrist [27]. The 2-year citation window used by JCR has been repeatedly criticized within the bibliometric community; there is a consensus that a citation window of only two years is far too short to calculate impact factor in many fields. For example, Huang and Lin argued that calculating journal impact factor over two years was too short and unfair to many disciplines, and proposed either a longer general citation window or different citation windows for different fields [28]. Other researchers thought that impact factor was a lagging indicator with a narrower time window; in many fields, citations accumulate slowly and the 2-year time window seems too short [29]. Campanario also argued that the two-year citation window was too brief to capture all relevant scientific impact [15]. In place of the shorter citation time window of 2IF, some scholars suggested using a longer citation window, while others proposed an evaluation indicator that could be used for any set of documents with any citation window. Dorta-Gonzalez and Dorta-Gonzalez wrote that, while in some cases two years was long enough to measure performance, in other cases, three or more years could be necessary [8]. Leydesdorff, Zhou & Bornmann pointed out that Thomson Reuters had extended the IF through a five-year variant (5IF) in response to criticism that a 2-year citation window might be too short for disciplines with slower turnovers [30]. Vanclay argued that, in social work, eight to ten years seemed a minimally appropriate time period [31]; this was consistent with recommendations to increase the citation window of Thomson ISI impact factors to ten years [32]. Leydesdorff & Bornmann proposed the I3 indicator (Integrated Impact Indicators) that could be used with any citation window [33]. Indicators for the Scopus database, such as SNIP and SJR, used a 3-year citation time window and a different definition of citable items [34].

To learn more about the evaluation effect of impact factors with different citation time windows, and to investigate the potential benefits of a longer citation time window, we conducted this study to provide a useful reference work for researchers in the field of bibliometrics.

## Methodology

### Research objects

There were 56 ophthalmologic journals in JCR-2012. We selected 28 journals with self-citation rates of less than 20% that were published by United States and indexed by the SCIE database between 2007 and 2012. Considering that ophthalmologic scholars in one country may not be familiar with journals published in other countries, we selected journals published in the United States to avoid scoring errors in the questionnaire survey. In addition, we selected journals with a self-citation rate of less than 20% so that excessive self-citation would not make a difference in the impact factors of various journals. In order to calculate journals’ impact factors using different citation time windows, we had to request journals indexed by SCIE database between 2007 and 2012 (for example: a journal’s 6IF in 2013 = citation in 2013 of papers published between 2007 and 2012/the number of citable documents published by the journal between 2007 and 2012).

### Research methods

#### Methods of calculating impact factor in 2013

Although the JCR database releases the 2IF and 5IF of journals annually, our studies also involved 3IF, 4IF, and 6IF, which couldn’t be obtained from the JCR database. For this reason, we had to calculate impact factors with different citation time windows by hand, using the citation analysis function of the Web of Science(WoS)database. Such computing methods have been explained in various documents [35–36]. For comparison purposes, 2IF and 5IF were also obtained by manual computation instead of using them released by the JCR.

#### Peer-reviewed scores of journals

We designed a survey questionnaire about the influence of different ophthalmologic journals on ophthalmologic researchers worldwide to accumulate peer-reviewed journal scores. Our survey on the influence of journals was designed to mirror their real status in the minds of researchers [37–38]. Harnad considered peer evaluation the most important recognized standard and method for verifying the validity of a citation index [39]. Considering subject classification in JCR database was more clear and obtained easily, we selected ophthalmologic journals and authors included by SCIE database as respondents. We thought those authors who published papers in ophthalmologic journals in SCIE database were researchers on the profession of ophthalmology and deeply understood their professional journals, thus they could evaluate journals’ impact from a professional point of view. In addition, to keep journals’ ranking from influencing questionnaire score, we ranked ophthalmologic journals in alphabetical order and told the investigators to give a score to each journal by their academic impact from 1.0 to 10.0 (the score is accurate to 1 decimal place, the “academic impact” in the questionnaire did not mean “impact factor” or any other indicator. It’s more like how useful and relevant the articles published in the journal were to author’ research, or how important the author thought the journal was in the field of ophthalmology). We got 8525 effective E-mail addresses of authors who published papers in SCIE database from 2008 to 2012 and sent 7742 questionnaires successfully. Finally, we got 291 responses and the response rate was 3.76% which was consistent with the result Shao Peiji [40] reported based on questionnaire survey of E-mail address. There were 239 valid responses and the rate of valid responses was 82.13%. All responses were from 36 countries and 61 of them were from American researchers. In the current study, we thoroughly processed the peer-reviewed results [41] of ophthalmologic journals in our questionnaires in 2012. As noted, all evaluation results related to American ophthalmologists and researchers reviewing ophthalmologic journals published in the United States, in order to avoid distorting our figures by asking peer-reviewed experts about unfamiliar journals from other countries or regions. Through this process, we received 61 valid responses from American scholars evaluating American journals. We calculated the total score for each journal as its final score. If a scholar left the section blank or said that a journal was ‘not familiar,’ such responses were equal to zero.

#### Ophthalmologic journals’ cited peak age

We retrieved papers published by 28 ophthalmologic journals for every year between 2001 and 2006. Through the citation analysis function of the WoS database, we analyzed changes in the number of citations for each year after papers were published. Finally we ascertained the peak age for citations of ophthalmologic journals, using the diachronic method[42].

#### Statistical process

SPSS 22.0 statistical software was used. Correlations between indicators were analyzed using the Spearman rank correlation test. Comparisons between impact factors with different citation time windows were made using the Kruskal-Wallis H test. The test level was alpha = 0.05.

## Results and Discussion

### Peer-reviewed scores of journals and impact factors with different citation time windows in 2013

Through this survey, we obtained the peer-reviewed scores of 28 ophthalmologic journals. At the same time, we calculated the journals’ impact factors within different citation time windows in 2013. These results were shown in Table 1.

**Table 1:** Peer-reviewed scores of 28 ophthalmologic journals and impact factors with different citation time windows in 2013.

Journal	Peer-reviewed scores	2IF	3IF	4IF	5IF	6IF
**Arch Ophthalmol**	411.8	4.357	4.217	4.486	4.304	4.191
**Invest Ophth Vis Sci**	401.4	3.508	3.620	3.623	3.626	3.785
**Am J Ophthalmol**	338.5	3.872	4.079	4.309	4.361	4.131
**Ophthalmology**	334.4	5.922	5.820	5.854	5.886	5.733
**Surv Ophthalmol**	237	3.466	3.456	3.582	3.659	3.745
**Exp Eye Res**	212	2.928	3.111	3.140	3.047	2.986
**Graef Arch Clin Exp**	192.5	2.149	2.114	2.181	2.186	2.099
**Retina-J Ret Vit Dis**	192	3.052	3.097	2.974	2.939	2.845
**Mol Vis**	164	2.101	2.224	2.253	2.303	2.239
**Curr Opin Ophthalmol**	162	2.671	2.844	2.843	2.823	2.829
**Cornea**	159.5	2.185	2.135	2.149	2.170	2.168
**J Cataract Refr Surg**	155.5	2.712	2.846	2.965	2.985	2.894
**J AAPOS**	140.5	1.117	1.199	1.209	1.246	1.197
**J Glaucoma**	134.5	1.879	1.935	2.020	2.095	2.091
**J Neuro-Ophthalmol**	121.5	1.835	1.584	1.575	1.481	1.460
**J Vision**	117	1.419	1.711	1.971	1.954	1.958
**Visual Neurosci**	107.5	1.635	1.700	1.940	1.690	1.616
**J Pediat Ophth Strab**	101.5	0.462	0.550	0.605	0.625	0.670
**J Ocul Pharmacol Th**	100.5	1.370	1.476	1.507	1.446	1.403
**Ophthal Plast Recons**	100	0.868	0.879	0.862	0.865	0.851
**Neuro-Ophthalmology**	97.5	0.216	0.211	0.213	0.209	0.189
**Ophthal Surg Las Im**	96.5	0.825	0.914	0.873	0.789	0.737
**J Refract Surg**	91.8	2.918	2.804	2.971	2.852	2.683
**Optometry Vision Sci**	79	1.824	2.021	2.123	2.160	2.192
**Ophthalmic Genet**	64.5	1.151	1.122	1.114	1.210	1.174
**Ocul Surf**	63	4.061	4.064	4.145	3.934	4.694
**Eye Contact Lens**	60	1.657	1.867	1.880	1.923	2.881
**Cutan Ocul Toxicol**	54.5	0.872	0.925	0.956	0.958	0.952
**Average**	160.4	2.251	2.304	2.369	2.347	2.361

As Table 1 demonstrates, nearly all journals with a higher peer-reviewed score had higher impact factors in the different citation time windows. The categorization of journals based on impact factors with different citation time windows was consistent. Sorting journals by their peer-reviewed scores produced results that were consistent with those achieved by studying impact factors with different citation time windows. The consistency in results produced by sorting journals using first, the peer-reviewed score and second, impact factors with different citation time windows suggested that it was reasonable to use impact factors with different citation time windows to evaluate journals.

### Correlations between peer-reviewed scores and impact factors with different citation time windows in 2013

**Fig 1:**
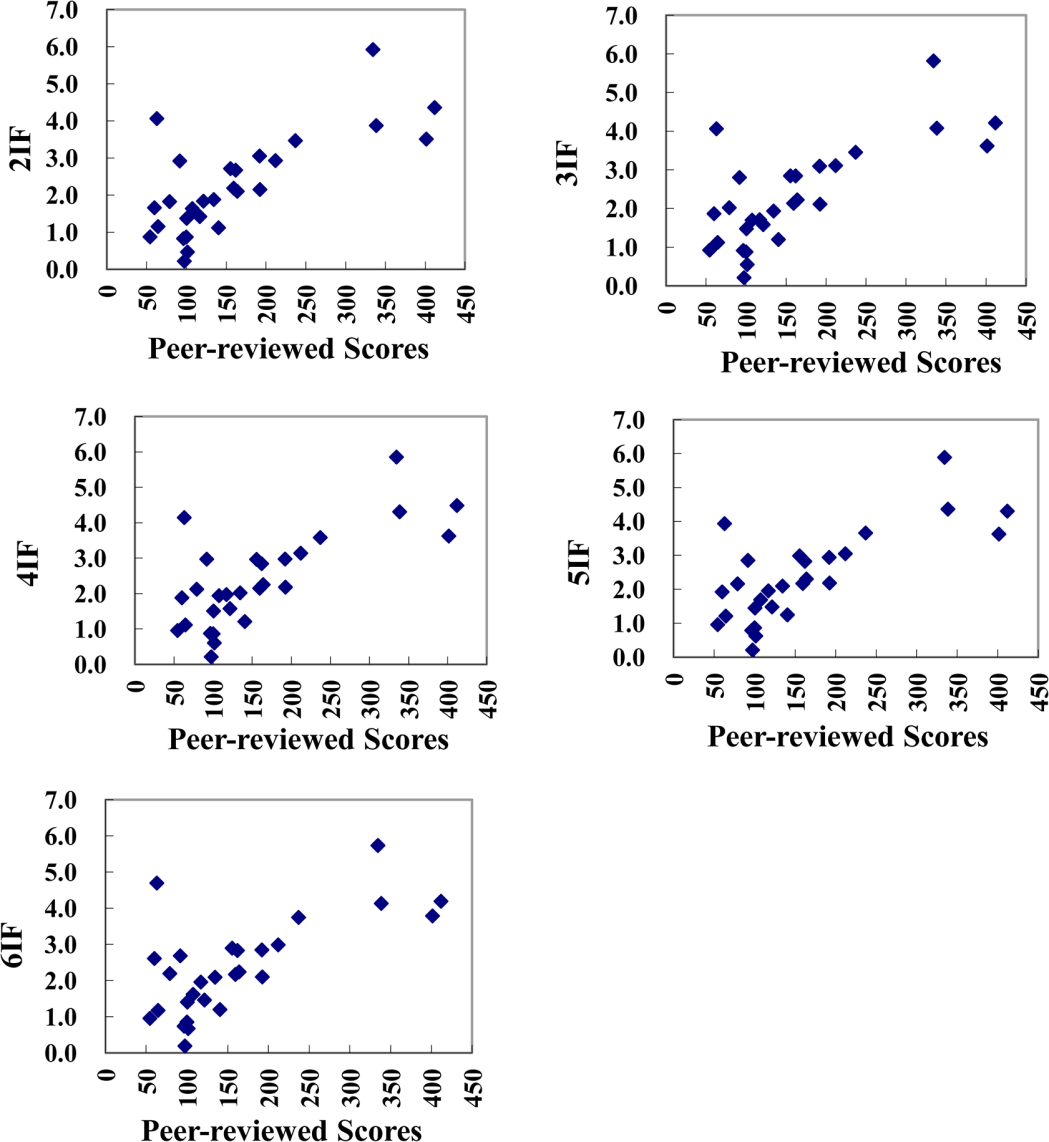
Correlations between the peer-reviewed scores of 28 ophthalmologic journals and impact factors with different citation time windows.

According to data shown in Table 1, we drew scatter diagrams of peer-reviewed scores of 28 ophthalmologic journals and their impact factors with different citation time windows in 2013 to further investigate the relationships between them (Fig 1), and no significant differences in Fig 1 are observed. The correlations between the journals’ peer-reviewed scores and impact factors with different citation time windows, as well as the correlations between impact factors with different citation time windows are illustrated in Table 2.

**Table 2:** Correlations between the peer-reviewed scores of 28 journals and impact factors with different citation time windows.

Test index	2IF	3IF	4IF	5IF	6IF
**Peer-reviewed scores**	0.652	0.667	0.667	0.664	0.585*
**2IF**		0.987	0.987	0.984	0.971
**3IF**			0.996	0.996	0.987
**4IF**				0.996	0.978
**5IF**					0.983

*P = 0.001, the others were *P*<0.001

In Table 2, note that peer-reviewed scores correlated well with all impact factors, regardless of which citation time window was used (all correlation coefficients between 0.58 and 0.67). In addition, correlations between 2IF, 3IF, 4IF, 5IF, and 6IF are all very high, with the lowest correlation coefficients being 0.971. It is easy to see that the evaluation results of 2IF, 3IF, 4IF, 5IF, and 6IF as journal evaluation indicators were highly consistent regardless of how their size changed.

### Comparisons between impact factors with different citation time windows

Considering that the distribution of impact factors with different citation time windows did not reflect normality, we chose, in the current study, to carry out a Kruskal-Wallis non-parametric test of 2IF, 3IF, 4IF, 5IF, and 6IF for 28 journals in 2013. The results were *χ* ^2^ = 0.640, *P* = 0.958, which indicates that the differences between the impact factors of 28 ophthalmologic journals with different citation time windows in 2013 were not significant.

In previous studies, many scholars [4,43] believed that 5IF was larger than 2IF for most journals, deducing that a long-term impact factor would be larger than a short-term impact factor for most journals. However, the results of the current study suggest that 4IF was the highest, followed by 6IF and 5IF; 2IF was genuinely the smallest. This doesn’t mean that the impact factor associated with a longer citation time window would be larger for all journals. Papers in some subjects quickly reached their cited peak, but in these cases, citations also tended to diminish fast. In other words, some documents aged very quickly. In these subjects, there may be cases of the impact factor associated with a longer citation time window being lower than that with a shorter window. Some scholars have suggested using different citation time windows to compute the impact factors of journals in different disciplines [3,8].

### Cited peak age of ophthalmologic papers

**Fig 2:**
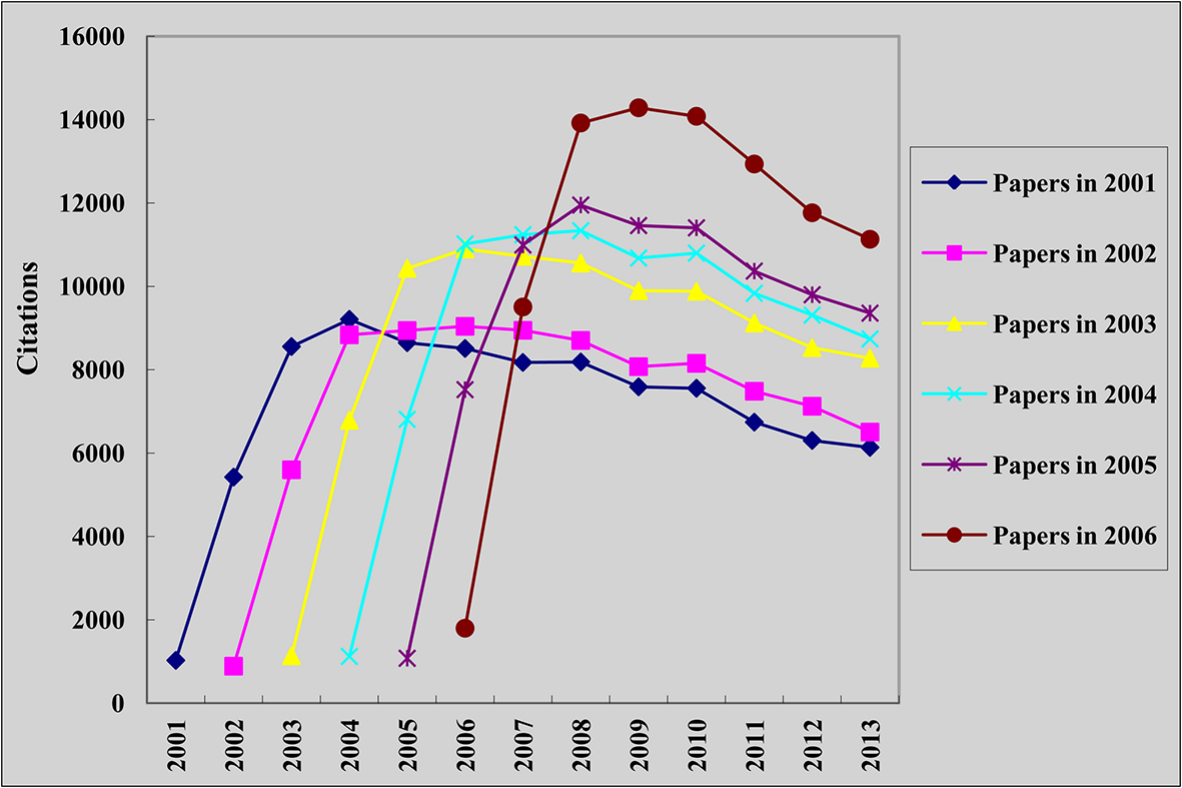
Citation evolution trends for papers published each year between 2001 and 2006 in 28 ophthalmologic journals.

We carried out an advanced retrieval type using the ISSN of 28 ophthalmologic journals. Next, we searched for papers each year between 2001 and 2006 and analyzed their citations for each successive year after the year of publication. In addition, we confirmed the cited peak age by observing the trends in citation evolution for published ophthalmologic papers. These results are shown in Fig 2.

As Fig 2 illustrates, citations in the first year after any year of publication were low, with the citation counts for all papers increasing rapidly both in the first and second years. The growth rate slowed down and citations reached their peak in the third year. Citations of papers published in 2003 reached their peak in 2006 and began to decline in the fourth year. Only the citations of papers published in 2004 reached their peak in the fourth year and began to decline in the fifth year. The results above are highly consistent with the finding that correlations between peer-reviewed scores and 3IF and 4IF were the highest in Table 2.

Della-Sala and others [14] at the University of Edinburgh in the U.K. concluded that the 2-year citation time window emphasized in recent studies was not very useful for evaluating journals in subjects whose cited peak came more slowly (in a slow-moving field). Hence, some scholars have argued for using an impact factor with a longer citation time window in journal evaluation. In 1998, Garfield [44–45] selected journals whose impact factor rankings were in the top 100 and 101–200 to calculate their 15IF and 7IF and to compare with one-year IF sorting. No great disparity was found in Garfield’s study, which was consistent with the current study finding that there are high correlations between impact factors with different citation time windows. Our study did not support that longer citation time windows were preferable; if anything, the correlation with peer-reviewed scores was somewhat less strong when the longest citation time window was used (6IF). Thus, for journals in a particular discipline, a longer citation time window may not be better for assessing impact factors.

## Conclusions

In this study, we used peer-reviewed journal rankings obtained through a survey as a gold standard to investigate the journal evaluation results of impact factors with different citation time windows. In general, in the field of ophthalmology, there were high correlations between journals’ peer-reviewed scores and impact factors with different citation time windows. There were also high correlations between impact factors with different citation time windows. It is clear that the length of a citation time window should be consistent with the cited peak.

As everyone is aware, different disciplines involve different properties and stages of development. For this reason, the values of impact factors with different citation time windows for journals in different subjects are necessarily different. However, by conducting empirical research within the field of ophthalmology, we found high correlations between 2IF, 3IF, 4IF, 5IF, and 6IF for evaluating journals.

In addition, we found high correlations between 2IF, 3IF, 4IF, 5IF, 6IF, and peer-reviewed scores for ophthalmologic journals in 2013, suggesting the scientific validity of using impact factors with different citation time windows to evaluate journals. For ophthalmologic journals it does not seem like longer citation time windows are preferable.

Our study results showed that the citation time window should be consistent with the cited peak of documents in a particular discipline. For example, if citations of documents published in a particular subject in (t) year reach their cited peak in (t+3) years, then the most appropriate citation time window would might be three years. For a particular subject, the length of the citation time window should be used to compute the impact factor in combination with the cited peak of the documents.

Finally, we must acknowledge the limitations of our study, in that we selected only ophthalmologic journals as research objects. The topic therefore should be investigated further to see whether other disciplines, in particular those with a later cited peak, followed the same or a similar pattern. In addition, Although citation peaks may follow a particular time window for individual journals, these citation time windows are very unlikely to remain fixed and may indeed be variable depending on the current events in a particular discipline, for these questions we will make further studies in the future.

## Supporting Information

S1 Dataset Data of questionnaire survey from American ophthalmologic researchers giving each American ophthalmology journal a score. (XLS)
